# Diagnostic Utility of Interleukin-6 in Early-Onset Sepsis among Term Newborns: Impact of Maternal Risk Factors and CRP Evaluation

**DOI:** 10.3390/children11010053

**Published:** 2023-12-30

**Authors:** Maria Schleier, Julia Lubig, Sven Kehl, Steven Hébert, Joachim Woelfle, Adriana van der Donk, Alisa Bär, Heiko Reutter, Tobias Hepp, Patrick Morhart

**Affiliations:** 1 Department of Pediatrics and Adolescent Medicine, Division of Neonatology and Pediatric Intensive Care, Friedrich-Alexander-University of Erlangen-Nürnberg, Loschgestraße 15, 91054 Erlangen, Germany; maria.schleier@fau.de (M.S.); julia.lubig@uk-erlangen.de (J.L.); steven.hebert@uk-erlangen.de (S.H.); joachim.woelfle@uk-erlangen.de (J.W.); adriana.vanderdonk@gmx.de (A.v.d.D.); alisa.baer@fau.de (A.B.); heiko.reutter@uk-erlangen.de (H.R.); 2 Department of Gynecology and Obstetrics Medicine, Division of Obstetrics, Friedrich-Alexander-University of Erlangen-Nürnberg, Universitätsstraße 21/23, 91054 Erlangen, Germany; sven.kehl@uk-erlangen.de; 3 Institute of Human Genetics, Friedrich-Alexander-University of Erlangen-Nürnberg, 91054 Erlangen, Germany; 4 Institute for Medical Informatics, Biometry and Epidemiology (IMBE), Friedrich-Alexander-University Erlangen-Nürnberg, Waldstraße 6, 91054 Erlangen, Germany; tbs.hepp@fau.de

**Keywords:** sepsis, neonatal, early-onset sepsis, EOS, Interleukin-6, C-reactive protein, newborn, risk factors, intrapartum maternal fever

## Abstract

(1) Background: Interleukin-6 (IL-6) levels act as an early infection marker preceding C-reactive protein (CRP) elevation. This study seeks to analyze IL-6 behavior in suspected early-onset sepsis (EOS) cases among term newborns, comparing it to that of CRP and evaluating IL-6’s diagnostic utility. We also aim to assess the impact of maternal risk factors on EOS in term newborns, quantifying their influence for informed decision making. (2) Methods: The retrospective data analysis included 533 term newborns who were admitted to our hospital because of suspected EOS. IL-6, CRP, and the impact of maternal risk factors were analyzed in the context of EOS using binomial test, Chi-squared test, logistic and linear regression. (3) Results: In the cases of EOS, both IL-6 and CRP were elevated. The increase in CRP can be predicted by the initial increase in IL-6 levels. Among the assessed risk factors, intrapartum maternal fever (adjusted odds ratio 18.1; 95% CI (1.7–4.1)) was identified as the only risk factor significantly associated with EOS. (4) Conclusions: Employing IL-6 as an early infection marker enhanced EOS diagnostic precision due to its detectable early rise. However, caution is required, as elevations in IL-6 and CRP levels do not exclusively indicate EOS. Increased CRP levels in healthy newborns with maternal risk factors may be attributed to dynamics of vaginal labor.

## 1. Introduction

Every year, approximately 1.4 million newborns worldwide die of infections [1]. Infections in the neonatal period include early-onset sepsis (EOS), which occurs in newborns within the first 72 h of life. Despite high standards of medical care, EOS still constitutes a dangerous complication, even in industrial countries [2,3].

In fall 2021, laboratory screening for detecting EOS at the Department of Pediatrics and Adolescent Medicine at Erlangen University Hospital was changed. Because of numerous studies showing the relevance of Interleukin-6 (IL-6) as an early laboratory parameter in cases of sepsis in the newborn period, IL-6 was established as a new laboratory marker [4,5,6]. In addition, clinical signs of sepsis were put into focus for diagnostics and assessment as newborns with maternal risk factors (such as Group B *Streptococcus* (GBS)-positive smear, intrapartum maternal fever, premature rupture (PROM) more than 18 h before birth) are now surveilled and screened by midwives and nurses for clinical signs (e.g., fever or tachycardia) of sepsis before a laboratory screening is performed. Contrastingly, maternal risk factors in combination with increased CRP levels, which were previously employed for EOS screening in all newborns, have since fall 2021 been utilized exclusively for EOS screening in newborns displaying sepsis symptoms.

The aim of this study was to evaluate the change in the diagnostic procedure focusing on clinical signs of sepsis and the establishment of the infection parameter IL-6 in laboratory screening. To facilitate a meaningful comparison between the years preceding and following the change in diagnostic assessment in fall 2021, the period from 2019 to 2022 was chosen as a suitable timeframe. Therefore, we retrospectively analyzed clinical data from term newborns admitted to our clinic due to suspected EOS in the period from January 2019 to December 2022.

## 2. Methods

### 2.1. Patients and Setting

We conducted an analysis of diagnostic and screening parameters for EOS using retro spective data from 533 term newborns (≥37 + 0 gestation age) treated in the Division of Neonatology at the Department of Pediatrics and Adolescent Medicine, Friedrich–Alexander-University of Erlangen-Nürnberg, due to suspected EOS between January 2019 and December 2022 (study population 1). In order to provide information on the influence of maternal risk factors for EOS, only those newborns born in the gynecological clinic of the University Hospital Erlangen were included in the analysis. Study population 2 consists of pregnant women who gave birth to 9496 term newborns in the gynecological clinic between January 2019 and December 2022 (study population 2). Clinical data were obtained from the clinical software systems Integrated Care Manager (version 12.01) by Dräger, Lauris (version 2.22.2) by Nexus/Swisslab, Soarian Clinicals (version 4.5.200) by Cerner, and ViewPoint 6 (version 6.12.3) by GE HealthCare.

### 2.2. Definitions

We defined sepsis based on the German AWMF guideline 024-008 ‘Bacterial infections in newborns’ [1] and on the NEO-KISS criteria of the German Neonatal Hospital Infection Surveillance System (referred as the ‘Official German’ sepsis classification) [7]. Sepsis can affect various systems of the body; depending on which system (e.g., respiratory, cardiovascular, metabolic) is affected, different symptoms arise [1,8,9,10,11,12]. The ‘Official German’ sepsis classification encompasses clinical and culture-proven sepsis.

To classify clinical sepsis, the following criteria must be met: (1) At least two clinical signs and symptoms of sepsis must be met (see below). (2) Antimicrobial therapy lasts for at least 5 days. (3) No bacterial growth is detected in blood culture, or no blood culture is taken. (4) No obvious infection is found elsewhere. 

To classify culture-proven sepsis, the following criteria must be met: (1) At least two clinical signs and symptoms of sepsis must be met (see below). (2) A pathogen (other than coagulase-negative *Staphylococcus*) is isolated from blood or cerebrospinal fluid.

Clinical signs and symptoms of sepsis used for classification at the University Hospital Erlangen:

- Fever (>38 °C/100.4 °F) or temperature instability or hypothermia (<36.5 °C/97.7 °F);
- Tachycardia (>200/min) or new/increased bradycardia (<80/min);
- Recurrent apneas (>20 s) or saturation drops;
- Prolonged recapillarization time >2 s or marbled skin coloration;
- Hypoglycemia (symptomatic < 45 mg/dL and asymptomatic < 35 mg/dL) or new onset hyperglycemia (>140 mg/dL);
- Unexplained metabolic acidosis (BE ≤ −10 mmoL/L); or
- Or at least one of the following signs of sepsis: C-reactive protein (CRP) >20 mg/L or Interleukin-6 (IL-6) >150 pg/mL; increased oxygen requirement [FiO_2_↑, intubation]; unstable general condition or poor drinking behavior; agitation; signs of respiratory distress;

The introduction of IL-6 at our laboratory took place in fall 2021. Until fall 2021, newborns without clinical signs of sepsis but with elevated CRP levels above 10 mg/L and an additional maternal risk factor had also been considered suspected EOS cases and received antibiotic therapy (referred as ‘Additional’ screening criteria). After changing the diagnostic procedure, respiratory distress symptoms that manifest immediately after birth have undergone more rigorous evaluation. If IL-6 and CRP test results are negative, these patients do not receive antibiotic therapy anymore. This was done to distinguish cases of breathing disorders unrelated to EOS. 

### 2.3. Diagnostic Procedure

**Before and after the diagnostic change:** Newborns meeting the ‘Official German’ guideline criteria for EOS received antibiotic therapy. Newborns whose clinical symptoms could not be assigned were treated with antibiotics as well until symptoms could be classified and a diagnosis was made. 

**Before the diagnostic change:** Additionally, a blood test measuring CRP levels was conducted on healthy newborns whose mothers had risk factors for EOS, such as intrapartum maternal fever, PROM > 18 h before birth, meconium-stained amniotic fluid, and a GBS-positive smear. Antibiotic therapy was initiated if the CRP levels of any of these newborns exceeded 10 mg/L (‘Additional’ screening criteria).

**After the diagnostic change:** CRP levels are not measured in healthy newborns with maternal risk factors anymore. Instead, newborns with a higher risk for EOS are now clinically monitored by midwives and nurses for up to 48 h after birth. At our hospital, meconium-stained amniotic fluid is no longer regarded as a risk factor for EOS due to recent studies that demonstrated its lack of independence as a risk factor [13,14]. Midwives document the newborns’ overall health, feeding behavior, heart and respiratory rates, skin condition, and temperature. If any concern arises, a physician is informed and will conduct a thorough examination, potentially ordering blood tests (IL-6 and CRP among other things) or blood cultures and initiating antibiotic therapy.

### 2.4. Study Population 1

Study population 1 (Table 1) consists of four groups.

**Group 1** is formed by 39 newborns (during the complete timeframe of the study) who were hospitalized because of suspected EOS and fulfilled the diagnostic ‘Official German’ EOS criteria (see Section 2.2).

**Group 2** is formed by 174 newborns (during the complete timeframe of the study) who were hospitalized because of suspected EOS showing clinical and often also laboratory signs of sepsis but did later not fulfill the ‘Official German’ criteria for EOS and were therefore diagnosed with mild neonatal infection.

**Group 3** is formed by 136 newborns (during the complete timeframe of the study) who showed respiratory signs of sepsis at hospitalization but were later classified as suffering from a breathing disorder not related to EOS. As of fall 2021, fewer newborns have fallen into this category, as treatment of respiratory distress was revised. If IL-6 and CRP test results are negative, these patients do not receive antibiotic therapy anymore.

**Group 4** is formed by 184 term newborns diagnosed with suspected EOS (until fall 2021) due to ‘Additional’ screening criteria for sepsis due to the former internal hospital guidelines. These patients presented elevated CRP levels above 10 mg/L and maternal risk factors for EOS (see Section 2.2) but no clinical symptoms.

All four groups were treated with antibiotics.

The following parameters were collected for each term newborn: sex, gestational age, mode of delivery, umbilical cord pH, age at admission, blood culture taken or not, duration of antibiotic therapy, respiratory support (invasive and noninvasive), clinical signs of infection at admission, and maternal risk factors for EOS. Laboratory chemistry analysis was supplemented with CRP and IL-6 measurements.

Newborns with the following characteristics were intentionally excluded from the study: externally born newborns, gestational age <37 + 0, umbilical cord pH at birth <7.0, major congenital anomalies, or incomplete data.

Newborns with incomplete data were excluded because their classification into categories of EOS or late-onset sepsis (LOS) and assignment to specific subgroups (groups 1–4) within study population 1 was not possible. Considering the low number of only 10 cases with incomplete data, these newborns were negligible.

### 2.5. Study Population 2 

In study population 2 (Table 2), all term newborns delivered at the Department of Obstetrics in the gynecological clinic between January 2019 and December 2022 were assessed for the impact of risk factors for sepsis originating from mothers. These maternal risk factors for EOS includes: GBS-positive smear, intrapartum maternal fever, PROM more than 18 h before birth, and meconium-stained amniotic fluid. Also, the mode of delivery, gestational age, and sex were recorded for each newborn. It was not possible to examine the distribution of the risk factors for 2019 and 2020 because the required detailed data were not available in the new software system of the Department of Obstetrics. Between January 2019 and December 2022, 9496 children were born at the university gynecological clinic.

### 2.6. Statistical Analysis 

The statistical analysis was conducted in collaboration with the Institute for Medical Informatics, Biometry, and Epidemiology (IMBE) of Friedrich–Alexander-University Erlangen-Nürnberg, Germany. All analyses were conducted using R version 4.1 (R Core Team 2023, Vienna, Austria) via the jamovi graphical user interface version 2.3.28 (The jamovi project 2023, Sydney, Australia). The relationship between IL-6 and CRP was analyzed with linear regression. The Chi-squared test was used for analyzing the effectivity of the new treatment in contrast to the old treatment for suspected EOS. The Binomial test was used to compare the delivery rate in group 4 with the delivery rate of the gynecological clinic. For evaluating the influence of the maternal risk factors, the Chi-squared test and binary logistic regression analysis were used. All *p*-values < 0.05 were considered statistically significant.

## 3. Results

### 3.1. EOS Cases

In total, 39 newborns received a diagnosis of sepsis during the entire period (2019–2022). In all 39 cases, the diagnosis of clinical sepsis was made, and in one of these cases, the blood culture also tested positive, leading to an additional diagnosis of culture-proven sepsis. In the latter instance, *Streptococcus agalacticae* (Group B beta-hemolytic *Streptococcus*) was identified in the blood culture. The newborn’s mother was tested negative for GBS before delivery and therefore did not receive antibiotic prophylaxis. The presence of coagulase-negative *Staphylococcus* in two blood cultures was considered contamination. Overall, the incidence for clinical sepsis in term newborns at our hospital was 4/1000 live births and for culture-proven sepsis was 0.1/1000 live births. Two newborns diagnosed with EOS also fulfilled the NEO-KISS criteria for pneumonia [7]. In 2022, our hospital did not experience an increase in escalated admissions related to EOS after the change in the diagnostic system in fall 2021 (see Table 1). Furthermore, the number of children diagnosed with LOS has remained consistent over the years. Specifically, in 2019, 3 cases of LOS; in 2020, 4 cases; in 2021, 4 cases; and in 2022, 1 case were recorded.

### 3.2. Relationship between IL-6 and CRP

For the full year of 2022, IL-6 levels of suspected EOS and EOS-classified newborns (67 cases) were examined in more detail in relation to the increase in CRP levels to assess whether IL-6 can be used as a reliable early infection marker. Four newborns with clinical sepsis who showed increased CRP levels above 10 mg/L (20.4 mg/L; 47.5 mg/L; 52.9 mg/L; 60.7 mg/L) and seven newborns with mild neonatal infection having CRP levels above 10 mg/L at admission were excluded. Their corresponding IL-6 increases probably occurred before admission. IL-6 levels above 150 pg/mL were considered abnormal. Observed IL-6 ranges in pg/mL for group 1 are min. 14, max. 6045, median 750; for group 2, min. 6, max. 51511, median 207; and for group 3, min. 9, max. 108, median 52.

Newborns with unremarkable CRP levels < 10 mg/L at admission and IL-6 levels above 150 pg/mL during their hospitalization showed an CRP increase of more than 5 mg/L in 59% of the cases and an CRP increase of more than 10 mg/L in 41% of the cases (mean CRP increase 15.7 mg/L).

CRP levels < 10 m/L at admission but IL-6 levels below 150 pg/mL showed a CRP-level increase of more than 5 mg/L in 11% of cases, with no cases of a CRP increase of more than 10 mg/L (mean CRP increase 1.6 mg/L).

Linear regression analysis was performed to estimate the effect of IL-6 on the differences between CRP at admission and the maximum observed value during the following three days (delta CRP). In order to meet the assumptions regarding linearity and normality of residuals, the natural logarithm of the IL-6 values was used. While similarly skewed, delta CRP was transformed using the square root due to the presence of zeros.

**Figure 1:**
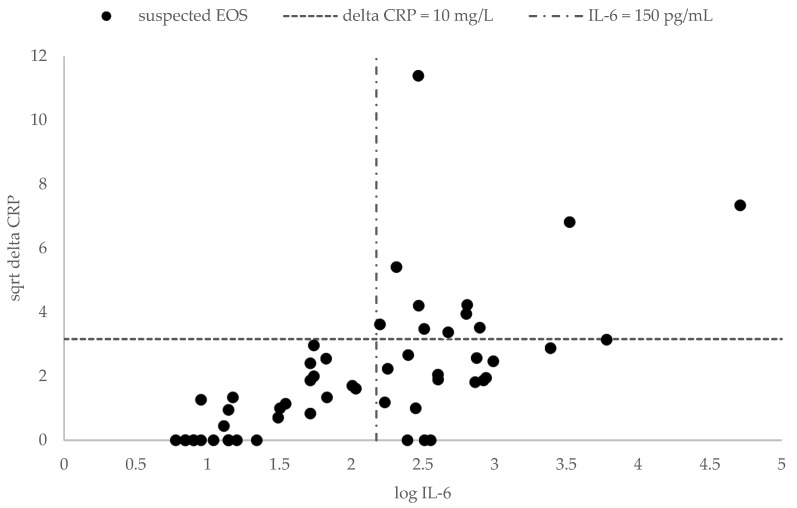
Scatterplot with log IL-6 (pg/mL) and delta CRP (mg/L). sqrt = square root.

Furthermore, the linear regression analysis proved statistically significantly (*p* < 0.001) that the increase in CRP can be predicted by observed IL-6 levels (Table 3). The scatter plot in Figure 1 illustrates the correlation between IL-6 (log IL-6) and the CRP-level increase (square root delta CRP).

### 3.3. Development of Case Numbers since Conversion of the Diagnostic Procedure

The evaluation showed that significantly fewer term newborns were treated because of neonatal sepsis when the new criteria for sepsis were applied after fall 2021. In relation to the baseline population (2019 and 2020 versus 2022), the number of term newborns treated for EOS decreased from 7.3% to 2.8% (*p* < 0.001) (Table 4). This means that in 2022, no newborns with maternal risk factors and CRP levels over 10 mg/L and without symptoms of sepsis required EOS treatment, compared to approximately 60–75 newborns per year who received antibiotic treatment in the preceding years (see Table 1, group 4). 

### 3.4. Analyzing Healthy Term Newborns with Maternal Risk Factors and Elevated CRP Levels above 10 mg/L

Patient group 4 consisting of 184 term newborns (having considered maternal risk factors for sepsis but no clinical signs of sepsis) showed high CRP levels with a mean value of 27.8 mg/L, median 24.2 mg/L, min. 10.0 mg/L, and max. 118 mg/L. The rate of vaginal delivery was significantly increased in this group to 84.8% (*p* < 0.001) compared to a rate of 74.4% vaginal deliveries in the population of the gynecological department (Table 5).

### 3.5. Evaluation of Maternal Risk Factors

Among the 18 cases of EOS diagnosed between 2021 and 2022, one newborn’s mother was GBS-positive, four mothers experienced intrapartum maternal fever, three had PROM lasting more than 18 h, and seven had meconium-stained amniotic fluid (Table 6). When considering each risk factor separately using the Chi-squared test, a significant effect was found for meconium-stained amniotic fluid (unadjusted odds ratio 3.13; *p* = 0.01) and intrapartum maternal fever (unadjusted odds ratio 22.2; *p* < 0.001). However, in binary logistic regression analysis of maternal risk factors associated with diagnosis of EOS, it was revealed that PROM more than 18 h before birth, maternal GBS-positive smear, and meconium-stained amniotic fluid were not significant at the 5% level for EOS. Meconium-stained amniotic fluid is therefore not an independent risk factor for EOS. A significant effect was only detected for the risk factor of intrapartum maternal fever (adjusted odds ratio (AOR) 18.1; *p* < 0.001) when considering these risk factors in combination (Table 7).

## 4. Discussion

The incidence of culture-proven EOS within our cohort of term newborns was 0.1 per 1000 live births. However, it is important to note that blood cultures were not obtained in all cases of clinical sepsis at our hospital, so there is the possibility that positive blood cultures may have been missed and the incidence might be slightly higher. The incidence of clinical EOS at our hospital was 4 per 1000 live births among term newborns. In comparison to culture-proven EOS rates reported in other studies, our population exhibits one of the lowest culture-proven EOS incidences. Research conducted in Thailand documented a rate of 0.22 per 1000 live births, which exhibits a similar rate compared to our results and also analyzed term newborns regarding EOS [15]. A study in the United Kingdom observed a rate of 0.7 per 1000 live births [16]. Similarly, a Swedish analysis reported a rate of 0.9 per 1000 live births [17], and US researchers observed a range of 0.77 to 0.98 per 1000 live births [3,13,18].

Several factors may account for the notable discrepancy in our findings compared to these studies. Our study had a precise focus solely on term newborns, encompassing those born at or after 37 weeks of gestation. Numerous other studies included a mix of both preterm and term infants, with culture-proven EOS incidence notably elevated in preterm infants [19,20]. This distinction is of particular relevance considering the impact of gestational age on sepsis occurrences. Furthermore, we exclusively considered EOS and did not incorporate instances of LOS. Additionally, our definition of EOS was confined to infections occurring within the initial 72 h following birth, rather than those extending beyond this timeframe.

The studies conducted by Pourcyrous et al. [21] and Sherwin et al. [22] have unveiled distinct temporal patterns in the behavior of IL-6 and CRP levels during neonatal sepsis. IL-6 concentrations exhibit their peak levels on the first day of neonatal sepsis, demonstrating a marked sensitivity within the initial hours of infection [21,22,23,24]. In contrast, CRP levels peak at a later point in time, typically around the second day, and can remain elevated up to five days [22]. This kinetic variation underscores the rapid turnover of IL-6, with a short half-life, rendering it highly responsive on the first day but progressively less on subsequent days of sepsis.

Our analysis revealed that IL-6 can effectively detect illness, including EOS and mild infections, in children. Therefore, IL-6 can serve as an early marker for identifying EOS and mild infections, offering a means to predict the subsequent elevation in CRP levels. Further investigations into the specificity and sensitivity of IL-6 could not be carried out due to the limited sample size of sepsis cases in 2022. IL-6 levels exhibited a consistent and reliable increase prior to the rise in CRP, offering a time-saving advantage of approximately one day in the diagnostic process for suspected EOS. A comparable diagnostic effect was also documented by Kuster et al., where elevated IL-6 levels were detected as much as two days prior to diagnosing LOS in preterm infants [6]. This underscores IL-6’s potential as a valuable tool to ascertain the likelihood of CRP elevation and therefore the probability of infection, particularly in patients undergoing regular blood tests. Measurements of IL-6 in the ICU, for both preterm and term infants, are valuable and could be conducted when a child is at an elevated risk of sepsis and shows signs of not being well, especially following major surgeries or as a result of multiple intravenous accesses. IL-6 may also prove beneficial for midwives in smaller gynecologic clinics lacking neonatology departments, enabling them to assess suspected infections or sepsis in cases where a child exhibits signs of infection.

However, the limitation of IL-6’s short half-life, especially for patients not admitted on the first day of sepsis, can be mitigated through the complementary consideration of CRP levels [25,26]. The kinetic interplay between CRP and IL-6 provides an approach to improve diagnostics. There were some measurements in our analysis where an increased IL-6 level was not followed by elevated CRP levels, which means there are some misleading positives.

Similar IL-6 levels were found for group 1 (EOS) and group 2 (mild infection). This shows that IL-6, while being a valuable early infection marker, does not offer definitive prediction regarding the intensity of infection in newborns—whether it constitutes a moderate infection or EOS. This is in line with the findings of Chiesa et al., who found that signs of sepsis are often subtle and unspecific and that IL-6 levels do not correlate with the symptoms developed later [27]. Different views on the sensitivity of IL-6 can be found in the literature [5,28,29,30,31,32]. The variability in the perspectives on IL-6 sensitivity depends on the specific study context. For instance, studies involving continuously monitored preterm infants in the neonatal ICU have a lower risk of missing IL-6 increases, as the monitoring is ongoing. Conversely, in the case of term newborns admitted with symptoms, IL-6 levels may have already risen by the time of admission. Furthermore, the choice of comparison groups in these studies can also influence the observed sensitivity of IL-6. Additionally, the diagnostic criteria used, such as the clinical sepsis definition or culture-proven definition, can impact the interpretation of IL-6’s sensitivity in diagnosing sepsis. An example given by the symptomatic neonate diagnosed with GBS in blood culture in our study without increased IL-6 and CRP levels shows that even laboratory markers may fail. So far, no single parameter for reliable prediction or surveillance of sepsis was found. Binnie et al. proposed that directing research attention toward sepsis epigenetics and the investigation of potential epigenetic markers and therapeutic targets holds promise for future studies [33].

Despite having elevated CRP levels above 10 mg/L, patients in group 4 did not show any signs of sepsis. This observation suggests that careful scrutiny and critical evaluation of elevated CRP levels in suspected EOS cases are essential, especially during the initial days of life. Our study not only reinforces this discovery but also underscores the importance of emphasizing it in clinical guidelines. The recognition of the potential variability and specific considerations surrounding CRP levels in the early days of life could contribute significantly to the refinement of diagnostic and treatment protocols in neonatal care. Previous research has underscored the presence of physiological CRP elevation in newborns [21,34,35]. For instance, Mjelle et al. discovered that approximately 1 in 14 healthy term newborns exhibited CRP levels exceeding 20 mg/L [35]. This physiological increase could be attributed to a range of factors, such as the influence of other conditions like active labor [36,37]. This observation finds support in studies conducted by Vogl et al. [38] and Gulmezoglu et al. [39], which suggest a correlation between stress during birth and elevated CRP levels. In our own analysis, we observed an increased percentage of vaginal delivery mode in group 4. This alignment with existing literature provides a plausible explanation for the elevated CRP levels observed within this subgroup. 

Concerns of physicians regarding inconspicuous infants with risk factors for EOS are common [14]. Consequently, these risk factors have often been considered important, despite the contention presented by several studies suggesting a more modest impact for some of them. Among these factors, intrapartum maternal fever emerged as highly significant in our study, exhibiting an AOR of 18.1, 95% CI (1.7–4.1). The ranking of intrapartum maternal fever matches with findings in other studies declaring a significant influence of fever with odds ratios of 2.38 and 4.05 [40,41].

GBS-positive smear showed no influence in our study. This is consistent with other studies where the GBS finding of the mother did not exhibit a discernible influence [14,42]. The insignificance of the GBS colonization can be attributed to the widespread use of antimicrobial prophylaxis provided to GBS-positive mothers at the onset of labor [43], with administration occurring at four-hour intervals until childbirth, as practiced at our hospital [44]. This prophylactic measure significantly reduces the transmission of maternal pathogens, subsequently decreasing the risk of colonization and infection in the newborn. Nevertheless, GBS continues to be the most frequently detected pathogen in blood cultures of term newborns with EOS [13].

In our study, PROM occurring more than 18 h before birth did not exhibit a statistically significant impact. (In order to facilitate a more comprehensive comparison of our findings, we additionally conducted tests regarding the rupture of membranes (ROM) occurring more than 18 h prior to birth. Our investigation revealed no statistically significant impact attributed to this risk factor. A detailed analysis concerning this matter has been appended for reference (Table A1).) Ofman et al. elucidates in their research that the absence of chorioamnionitis negates the influence of PROM [45]. Conflicting findings can be found in other studies, where a substantial influence has been observed in this context [13,15,40,41]. In cases where opportunities for preventing GBS infection are missed, such as the failure to screen and provide antibiotics to GBS-positive mothers, PROM can have a significant impact [13,15]. 

The fourth examined risk factor, meconium-stained amniotic fluid, also displayed a lack of statistically significant influence on EOS within our study. Notably, Cortés’s and Escobar’s investigations yielded a significant influence for meconium-stained amniotic fluid, with odds ratios of 9.04 and 2.23 among newborns [5,46]. However, divergent conclusions emerge in literature; Poupolo et al. [14] assert that meconium-stained amniotic fluid is not an independent predictor for EOS, while the work of Stoll et al. [13] also aligns with these findings. In our study, when we initially assessed the risk of meconium-stained amniotic fluid using unadjusted odds ratios, it appeared to have a statistically significant impact on EOS. However, when we conducted logistic regression analysis considering other risk factors in the model, we did not observe a significant influence of meconium-stained amniotic fluid. This suggests that meconium-stained amniotic fluid is not an independent risk factor for EOS, and it underscores the importance of employing logistic regression analysis to account for confounding variables, which was not done in the study by Cortes et al. [5]. Additionally, studies solely assessing maternal risk factors may yield different results from those that also incorporate infant factors like the infant’s appearance and absolute neutrophil count. The inclusion of infant factors can result in varying levels of influence for meconium-stained amniotic fluid, as demonstrated in the study conducted by Escobar et al. [46].

Maternal risk factors, particularly intrapartum maternal fever, play a role in EOS cases and should be diligently documented in the corresponding medical files. A proven noninvasive approach practiced in our hospital involves monitoring of the newborns’ health up to 48 h after birth by midwives and nurses (see Section 2.3.). Obstetric midwives and nurses should receive instruction for clinical surveillance and reporting of clinically concerning cases to physicians.

When considering the entire year 2022, no escalated numbers of admissions related to EOS or LOS were observed at our hospital. This outcome underscores the efficacy of the diagnostic modifications in preventing the administration of unnecessary antibiotics to children. This alignment of findings can be found in the work of Kiser et al., who assert that treating asymptomatic newborns with CRP levels around 10 mg/L with antibiotics would result in overtreatment [47]. Correspondingly, Escobar et al. echo these sentiments, highlighting a low risk of EOS in asymptomatic newborns [46].

Our study has the following limitations: (1) The study relies on retrospective clinical and microbiological data. The data collection was initially not established with the intention of conducting this study, leading to partial incompleteness of patient information. (2) Due to the absence of a standardized definition for clinical sepsis and diagnosis being based on individual, subjective classification by the physician, comparing numbers between studies might be challenging. (3) Because of the small size of the study cohort and the fact that it is a single-center study, the value of the findings might be limited and not easily transferable to other institutions. (4) Blood cultures were not obtained in all cases, which leaves us without the information that these cultures might have provided. Considering the constrained blood volume that can be drawn from neonates due to their physical condition (often as little as one milliliter of blood), there is a possibility that this limitation could have contributed to an increased likelihood of false negative results. (5) Given the high standard of medical care in our study population, the risk of EOS might be lower than usual, thus potentially limiting the generalizability of the results to countries with similar medical and hygienic standards. (6) The carrier status for GBS was not assessed in all births, resulting in a lack of information. (7) Given that the exact duration of labor was not documented, we can only assume the relation between dynamic/prolonged vaginal labor and elevated CRP levels in newborns.

## 5. Conclusions

Our data show that IL-6 represents a suitable early infection marker for laboratory screening in suspected cases of neonatal EOS, given its detectable early rise. The shifts in diagnostic procedures concerning risk factors for EOS within our clinic were considered valuable and proven to be statistically significant. Within this context, a discernible impact could be established only for intrapartum maternal fever (AOR 18.1; *p* < 0.001), while elevated CRP levels observed in healthy newborns with maternal risk factors might find explanation in the dynamics of vaginal labor. Our findings support the high sensitivity and specificity of detecting EOS by focusing on clinical abnormalities followed by the combined determination of CRP and IL-6. Routine blood screening tests should not be conducted for term newborns with maternal risk factors for EOS that do not show clinical symptoms of EOS, as this can lead to a high rate of false-positive CRP results. Switching to an approach of monitoring newborns with risk factors for EOS by midwives and ordering blood tests (IL-6 and CRP) only when the neonates exhibit any symptoms associated with EOS showed excellent results. This approach reduced the number of term newborns treated unnecessarily in our unit significantly by 60 to 75 newborns per year, while the numbers of LOS cases remained stable.

## Figures and Tables

**Table 1:** Overview study population 1 (Neonatology Division).

		Year				
Study Population 1	Definition	2019	2020	2021	2022	Total
Group 1	Early-onset sepsis (EOS)	14	7	7	11	39
Group 2	Mild infection	39	54	39	42	174
Group 3	Breathing disorder not related to EOS	40	54	28	14	136
Group 4	Laboratory abnormal, clinically unremarkable	74	62	48	0	184
All groups		167	177	122	67	533

**Table 2:** Study population 2: term newborns and their maternal risk factors for EOS, along with the vaginal delivery rates in the gynecological clinic. Numbers in brackets note the percentage per year.

	Year				
Study Population 2	2019	2020	2021	2022	Total
Term Newborns	2297 (100)	2388 (100)	2438 (100)	2373 (100)	9496
GBS-positive smear	-	-	186 (7.6)	188 (7.9)	374
Intrapartum maternal fever	-	-	30 (1.2)	35 (1.5)	65
PROM > 18 h	-	-	384 (15.8)	377 (15.9)	761
Meconium-stained amniotic fluid	-	-	435 (17.8)	382 (16.1)	817
Vaginal delivery	1737 (75.6)	1757 (73.6)	1830 (75.1)	1738 (73.2)	7062

**Table 3:** Linear regression analysis with square root delta CRP (mg/L) and log IL-6 (pg/mL).

Coefficient–Square Root Delta CRP				
Predictor	Estimate	Std. Error	t	*p*
Log IL-6	1.59	0.263	6.05	<0.001

**Table 4:** Contingency table and Chi-squared test with the number of newborns treated because of (suspected) EOS in relation to the number of term newborns born in the gynecological clinic.

	Term Newborns in the Gynecological Clinic		
Treated Because of (Suspected) EOS	2019 and 2020	2022	Total
Yes	344	67	411
No	4341	2306	6647
Total	4685	2373	7058
χ²-Test	Value	df	*p*
χ²	58.7	1	<0.001
N	7058		

**Table 5:** Binomial test performed for vaginal delivery versus non-vaginal delivery (primary, secondary, and emergency cesarean section) in the group of term newborns treated because of elevated CRP levels above 10 mg/L having maternal risk factors at birth but showing no signs of sepsis. * Results significant at a 95% confidence interval.

						95% CI	
Binomial Test	Level	Count	Total	Proportion	*p*	Lower	Upper
Delivery mode	Non-vaginal delivery	28	184	0.15	<0.001 *	0.1	0.21
	Vaginal delivery	156	184	0.85	<0.001 *	0.79	0.9

Note: Hₐ is a proportion ≠ 0.744.

**Table 6:** The numbers of sepsis and non-sepsis cases for each maternal risk factor from 2021 to 2022.

	Sepsis	Non-Sepsis	Total Number of Children with Risk Factors
GBS-positive smear	1	373	374
Intrapartum maternal fever	4	61	65
PROM > 18 h	3	758	761
Meconium-stained amniotic fluid	7	810	817

**Table 7:** Logistic regression analysis and Chi-squared test performed for maternal risk factors for EOS. * Results significant at a 95% confidence interval.

	95% CI						
Predictor for EOS	Lower	Upper	Std. Error	*p*	Adjusted Odds Ratio	*p*	Unadjusted Odds Ratio
GBS-positive smear	−2.50	1.56	1.04	0.65	0.63	0.73	0.69
Intrapartum maternal fever	1.70	4.09	0.61	<0.001 *	18.1	<0.001 *	22.2
PROM > 18 h	−1.48	1.07	0.65	0.75	0.81	0.92	1.06
Meconium-stained amniotic fluid	−0.13	1.86	0.51	0.09	2.38	0.01 *	3.13

## Data Availability

The datasets analyzed in this study are available from the corresponding author on request. The data are not publicly available due to privacy or ethical restrictions.

## Appendix A

**Table A1:** Logistic regression analysis performed for maternal risk factors for EOS. Logistic regression analysis considering ROM > 18 h. * Results significant at a 95% confidence interval.

95% CI					
Predictor for EOS	Lower	Upper	Std. Error	*p*	Adjusted
					Odds Ratio
GBS-positive smear	−2.49	1.58	1.04	0.66	0.63
Intrapartum maternal fever	1.77	4.18	0.61	<0.001 *	19.5
ROM > 18 h	−1.84	0.74	0.66	0.4	0.58
Meconium-stained amniotic fluid	−0.1	1.89	0.51	0.08	2.45

## References

[1] AWMF Bakterielle Infektionen bei Neugeborenen. https://register.awmf.org/assets/guidelines/024-008l_S2k_Bakterielle_Infektionen_Neugeborene_2021-03.pdf.

[2] Wynn J.L. (2016). Defining neonatal sepsis. Curr. Opin. Pediatr..

[3] Weston E.J., Pondo T., Lewis M.M., Martell-Cleary P., Morin C., Jewell B., Daily P., Apostol M., Petit S., Farley M. (2011). The burden of invasive early-onset neonatal sepsis in the United States, 2005–2008. Pediatr. Infect. Dis. J..

[4] Su H., Chang S.S., Han C.M., Wu K.Y., Li M.C., Huang C.Y., Lee C.L., Wu J.Y., Lee C.C. (2014). Inflammatory markers in cord blood or maternal serum for early detection of neonatal sepsis-a systemic review and meta-analysis. J. Perinatol..

[5] Cortes J.S., Losada P.X., Fernandez L.X., Beltran E., DeLaura I., Narvaez C.F., Fonseca-Becerra C.E. (2021). Interleukin-6 as a Biomarker of Early-Onset Neonatal Sepsis. Am. J. Perinatol..

[6] Kuster H., Weiss M., Willeitner A.E., Detlefsen S., Jeremias I., Zbojan J., Geiger R., Lipowsky G., Simbruner G. (1998). Interleukin-1 receptor antagonist and interleukin-6 for early diagnosis of neonatal sepsis 2 days before clinical manifestation. Lancet.

[7] Nationales Referenzzentrum für Surveillance von nosokomialen Infektionen am Institut für Hygiene und Umweltmedizin Charité—Universitätsmedizin Berl Protokoll Surveillance von Nosokomialen Infektionen, Multiresistenten Erregern und Antibiotika-Anwendungen bei Frühgeborenen mit Einem Geburtsgewicht unter 1500g. https://www.nrz-hygiene.de/files/Protokolle/NEO%20Protokolle/NEOKISS_Protokoll_Jun_2020.pdf.

[8] Ohlin A., Bjorkqvist M., Montgomery S.M., Schollin J. (2010). Clinical signs and CRP values associated with blood culture results in neonates evaluated for suspected sepsis. Acta Paediatr..

[9] Bohnhorst B., Lange M., Bartels D.B., Bejo L., Hoy L., Peter C. (2012). Procalcitonin and valuable clinical symptoms in the early detection of neonatal late-onset bacterial infection. Acta Paediatr..

[10] Celik I.H., Hanna M., Canpolat F.E., Mohan P. (2022). Diagnosis of neonatal sepsis: The past, present and future. Pediatr. Res..

[11] Young Infants Clinical Signs Study G. (2008). Clinical signs that predict severe illness in children under age 2 months: A multicentre study. Lancet.

[12] The WHO Young Infants Study Group (1999). Clinical prediction of serious bacterial infections in young infants in developing countries. Pediatr. Infect. Dis. J..

[13] Stoll B.J., Hansen N.I., Sanchez P.J., Faix R.G., Poindexter B.B., Van Meurs K.P., Bizzarro M.J., Goldberg R.N., Frantz I.D., Hale E.C. (2011). Early onset neonatal sepsis: The burden of group B Streptococcal and E. coli disease continues. Pediatrics.

[14] Puopolo K.M., Benitz W.E., Zaoutis T.E., Cummings J., Juul S., Hand I., Eichenwald E., Poindexter B., Committee on Fetus and Newborn, Committee on Infectious Diseases (2018). Management of Neonates Born at >/=35 0/7 Weeks’ Gestation with Suspected or Proven Early-Onset Bacterial Sepsis. Pediatrics.

[15] Aeimcharnbanchong K. (2023). Incidence Rate and Associated Factors of Early Onset Sepsis Among Neonate Born at >/=35 Weeks’ Gestation in Thai Tertiary Hospital. Infect. Drug Resist..

[16] Cailes B., Kortsalioudaki C., Buttery J., Pattnayak S., Greenough A., Matthes J., Russell A.B., Kennea N., Heath P.T. (2018). Epidemiology of UK neonatal infections: The neonIN infection surveillance network. Arch. Dis. Child Fetal. Neonatal. Ed..

[17] Johansson Gudjonsdottir M., Elfvin A., Hentz E., Adlerberth I., Tessin I., Trollfors B. (2019). Changes in incidence and etiology of early-onset neonatal infections 1997-2017 - a retrospective cohort study in western Sweden. BMC Pediatr..

[18] Schrag S.J., Farley M.M., Petit S., Reingold A., Weston E.J., Pondo T., Hudson Jain J., Lynfield R. (2016). Epidemiology of Invasive Early-Onset Neonatal Sepsis, 2005 to 2014. Pediatrics.

[19] Bailit J.L., Gregory K.D., Reddy U.M., Gonzalez-Quintero V.H., Hibbard J.U., Ramirez M.M., Branch D.W., Burkman R., Haberman S., Hatjis C.G. (2010). Maternal and neonatal outcomes by labor onset type and gestational age. Am. J. Obstet. Gynecol..

[20] Flannery D.D., Puopolo K.M. (2022). Neonatal Early-Onset Sepsis. Neoreviews.

[21] Pourcyrous M., Korones S.B., Crouse D., Bada H.S. (1998). Interleukin-6, C-reactive protein, and abnormal cardiorespiratory responses to immunization in premature infants. Pediatrics.

[22] Sherwin C., Broadbent R., Young S., Worth J., McCaffrey F., Medlicott N.J., Reith D. (2008). Utility of interleukin-12 and interleukin-10 in comparison with other cytokines and acute-phase reactants in the diagnosis of neonatal sepsis. Am. J. Perinatol..

[23] Hedegaard S.S., Wisborg K., Hvas A.M. (2015). Diagnostic utility of biomarkers for neonatal sepsis--a systematic review. Infect. Dis..

[24] Martin H., Olander B., Norman M. (2001). Reactive hyperemia and interleukin 6, interleukin 8, and tumor necrosis factor-alpha in the diagnosis of early-onset neonatal sepsis. Pediatrics.

[25] Arnon S., Litmanovitz I. (2008). Diagnostic tests in neonatal sepsis. Curr. Opin. Infect. Dis..

[26] Celik I.H., Demirel F.G., Uras N., Oguz S.S., Erdeve O., Biyikli Z., Dilmen U. (2010). What are the cut-off levels for IL-6 and CRP in neonatal sepsis?. J. Clin. Lab. Anal..

[27] Chiesa C., Pellegrini G., Panero A., Osborn J.F., Signore F., Assumma M., Pacifico L. (2003). C-reactive protein, interleukin-6, and procalcitonin in the immediate postnatal period: Influence of illness severity, risk status, antenatal and perinatal complications, and infection. Clin. Chem..

[28] Eichberger J., Resch B. (2022). Reliability of Interleukin-6 Alone and in Combination for Diagnosis of Early Onset Neonatal Sepsis: Systematic Review. Front. Pediatr..

[29] Magudumana M.O., Ballot D.E., Cooper P.A., Trusler J., Cory B.J., Viljoen E., Carter A.C. (2000). Serial interleukin 6 measurements in the early diagnosis of neonatal sepsis. J. Trop. Pediatr..

[30] Froeschle G.M., Bedke T., Boettcher M., Huber S., Singer D., Ebenebe C.U. (2021). T cell cytokines in the diagnostic of early-onset sepsis. Pediatr. Res..

[31] Ebenebe C.U., Hesse F., Blohm M.E., Jung R., Kunzmann S., Singer D. (2021). Diagnostic accuracy of interleukin-6 for early-onset sepsis in preterm neonates. J. Matern. Fetal. Neonatal. Med..

[32] Steinberger E., Hofer N., Resch B. (2014). Cord blood procalcitonin and Interleukin-6 are highly sensitive and specific in the prediction of early-onset sepsis in preterm infants. Scand. J. Clin. Lab. Investig..

[33] Binnie A., Tsang J.L.Y., Hu P., Carrasqueiro G., Castelo-Branco P., Dos Santos C.C. (2020). Epigenetics of Sepsis. Crit. Care. Med..

[34] Ainbender E., Cabatu E.E., Guzman D.M., Sweet A.Y. (1982). Serum C-reactive protein and problems of newborn infants. J. Pediatr..

[35] Mjelle A.B., Guthe H.J.T., Reigstad H., Bjorke-Monsen A.L., Markestad T. (2019). Serum concentrations of C-reactive protein in healthy term-born Norwegian infants 48-72 hours after birth. Acta Paediatr..

[36] Kaapa P., Koistinen E. (1993). Maternal and neonatal C-reactive protein after interventions during delivery. Acta Obstet. Gynecol. Scand..

[37] Ishibashi M., Takemura Y., Ishida H., Watanabe K., Kawai T. (2002). C-reactive protein kinetics in newborns: Application of a high-sensitivity analytic method in its determination. Clin. Chem..

[38] Vogl S.E., Worda C., Egarter C., Bieglmayer C., Szekeres T., Huber J., Husslein P. (2006). Mode of delivery is associated with maternal and fetal endocrine stress response. BJOG.

[39] Gulmezoglu A.M., Mahomed K., Hofmeyr G.J., Nikodem V.C., Kramer T. (1996). Fetal and maternal catecholamine levels at delivery. J. Perinat. Med..

[40] Puopolo K.M., Draper D., Wi S., Newman T.B., Zupancic J., Lieberman E., Smith M., Escobar G.J. (2011). Estimating the probability of neonatal early-onset infection on the basis of maternal risk factors. Pediatrics.

[41] Benitz W.E., Gould J.B., Druzin M.L. (1999). Risk factors for early-onset group B streptococcal sepsis: Estimation of odds ratios by critical literature review. Pediatrics.

[42] Benitz W.E., Wynn J.L., Polin R.A. (2015). Reappraisal of guidelines for management of neonates with suspected early-onset sepsis. J. Pediatr..

[43] Wicker E., Lander F., Weidemann F., Hufnagel M., Berner R., Krause G. (2019). Group B Streptococci: Declining Incidence in Infants in Germany. Pediatr. Infect. Dis. J..

[44] AWMF Prophylaxe der Neugeborenensepsis - Frühe Form - durch Streptokokken der Gruppe B. https://register.awmf.org/assets/guidelines/024-020l_S2k_Prophylaxe_Neugeborenensepsis_Streptokokken_2016-04-abgelaufen.pdf.

[45] Ofman G., Vasco N., Cantey J.B. (2016). Risk of Early-Onset Sepsis following Preterm, Prolonged Rupture of Membranes with or without Chorioamnionitis. Am. J. Perinatol..

[46] Escobar G.J., Li D.K., Armstrong M.A., Gardner M.N., Folck B.F., Verdi J.E., Xiong B., Bergen R. (2000). Neonatal sepsis workups in infants >/=2000 grams at birth: A population-based study. Pediatrics.

[47] Kiser C., Nawab U., McKenna K., Aghai Z.H. (2014). Role of guidelines on length of therapy in chorioamnionitis and neonatal sepsis. Pediatrics.

